# Partial Laryngectomy with Cricoid Reconstruction: Thyroid Carcinoma Invading the Larynx

**DOI:** 10.1155/2014/671902

**Published:** 2014-02-10

**Authors:** Kerem Ozturk, Serdar Akyildiz, Ozer Makay

**Affiliations:** ^1^Department of Otolaryngology, Ege University School of Medicine, Bornova, 35100 Izmir, Turkey; ^2^Department of General Surgery, Ege University School of Medicine, Bornova, 35100 Izmir, Turkey

## Abstract

Laryngotracheal invasion worsens the prognosis of thyroid cancer and the surgical approach for laryngotracheal invasion is controversial. In this paper, partial full-thickness excision of the cricoid cartilage with supracricoid laryngectomy and reconstruction of existing defect with thyroid cartilage are explained in a patient with papillary thyroid carcinoma invading the thyroid cartilage and cricoid cartilage without intraluminal invasion. Surgical indication should not be established by the site of involvement in thyroid carcinomas invading the larynx, as in primary cancers of the larynx. We think that partial laryngectomy according to the involvement site and the appropriate reconstruction techniques should be used for thyroid cancer invading the larynx.

## 1. Introduction

Well-differentiated thyroid cancers are low-grade malignancies with a good prognosis. Ten-year survival is over 80%. However, a small part of these cancers has an aggressive character and jeopardizes the patient's life by infiltrating surrounding structures [1]. Although the rate of laryngotracheal invasion of differentiated thyroid carcinoma is about 7% [2], less than 1% infiltrates the lumen [3]. The vast majority of deaths depend on the involvement of surrounding organs or distant metastases. Therefore, local control of the disease is effective on survival rate [4]. Laryngotracheal invasion worsens the prognosis of thyroid cancer and the surgical approach for laryngotracheal invasion is controversial [5]. The more radical the surgical technique used is, the more the patient's quality of life will be effected [4]. In this paper, partial full-thickness excision of the cricoid cartilage with supracricoid laryngectomy and reconstruction of existing defect with laryngeal cartilage are explained in a patient with papillary thyroid carcinoma invading the thyroid cartilage and cricoid cartilage without intraluminal invasion.

## 2. Case Report

**Figure 1:**
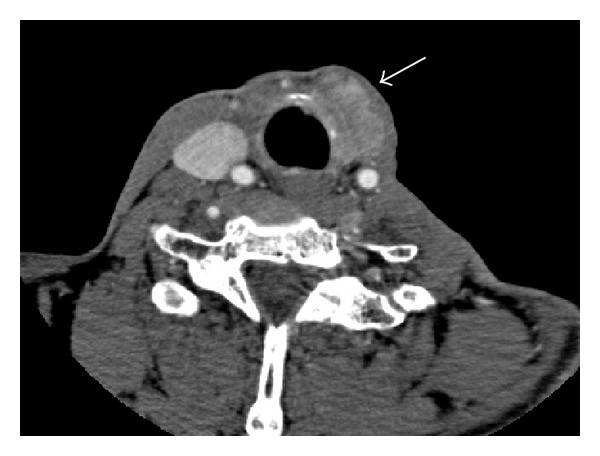
CT scan showing cricoid cartilage invasion by thyroid carcinoma (arrow).

A 50-year-old male patient who underwent total thyroidectomy and neck dissection with the diagnosis of thyroid papillary carcinoma 17 years ago presented to our clinic with the complaint of swelling in a region corresponding to the thyroid compartment on the left side. A 4 × 3 cm sized hard mass was palpated on the left side at the level of cricoid on physical examination. Endoscopic examination of the larynx and vocal cord movements were normal. Ultrasonographic examination revealed a solid mass with heterogeneous internal structure that was about 4.5 × 2.5 × 4 cm in size invading the laryngeal structure. Fine-needle aspiration biopsy taken from the mass was found to be compatible with the thyroid papillary carcinoma. Neck CT showed a malignant mass lesion extending to the larynx in the cranial direction in the thyroid compartment and destructing cricoid cartilage and thyroid cartilage (Figure 1). The patient was scheduled for a laryngectomy and postoperative radioactive iodine based on these findings.

**Figure 2:**
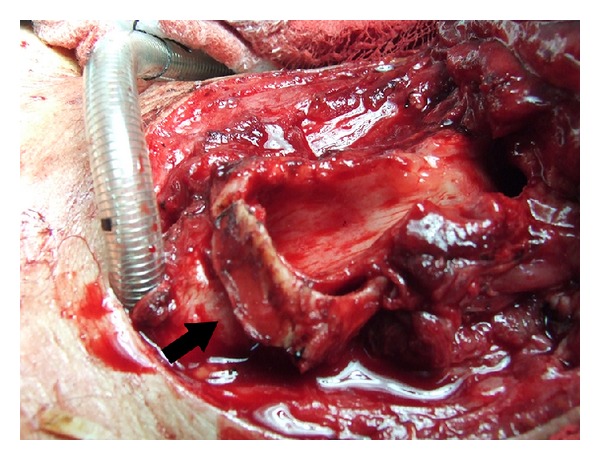
Using thyroid cartilage for cricoid reconstruction (arrow) (before suturation).

**Figure 3:**
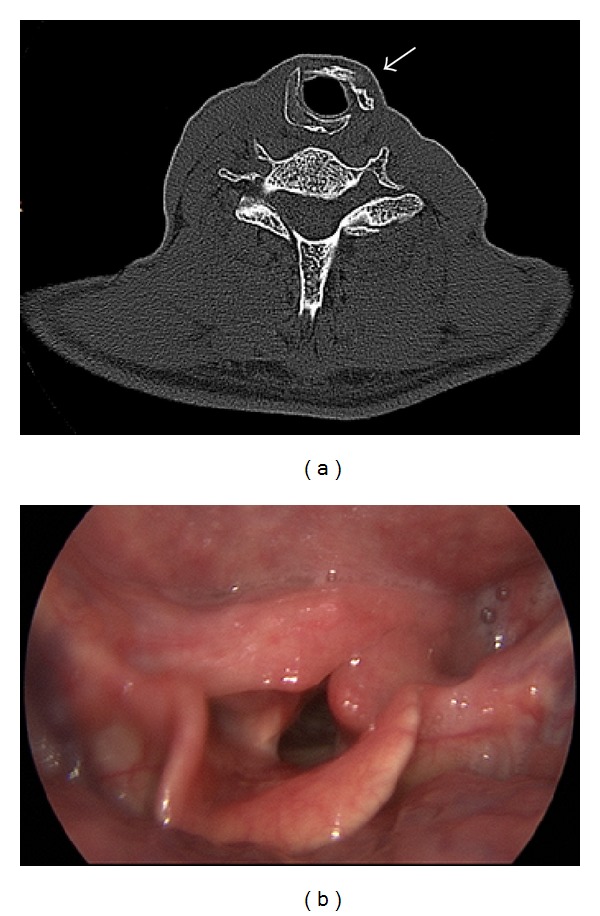
(a) CT scan showing thyroid cartilage used for reconstruction (arrow). (b) Videolaryngoscopic image showing adequate airway passage.

Under general anesthesia, the previous operative thyroidectomy incision was entered and skin flaps were elevated. After the excision of infrahyoid muscles invaded by tumor tissue, the larynx was reached. Left half of the thyroid cartilage and the region towards the left side from the midline of cricoid cartilage up to 5 mm in front of cricoarytenoid cartilage were found to be invaded by the tumor. When the left half of the thyroid cartilage was elevated, left paraglottic space adjacent to cartilage was found to be minimally involved. The patient first underwent supracricoid laryngectomy. Full thickness excision of invaded part of cricoid cartilage was performed by preserving inner mucosa. The upper right half of the thyroid cartilage not invaded by tumor including the superior cornu was shaped and sutured to the site of the resected part of the cricoid, and cricoid framework was recreated and protected inner mucosa was mounted in this framework (Figure 2). The larynx was closed by cricohyoidoepiglottopexy. There were no complications postoperatively. The nasogastric tube was removed on the 16th postoperative day and the patient was decannulated on the 17th postoperative day. The result of the pathological examination was papillary carcinoma invasion of the thyroid cartilage and cricoid cartilage. Then radioactive iodine treatment was given. Narrowing of the laryngeal air passage and local recurrence were not detected during the 3-year follow-up period. The control CT scan of the neck has shown no evidence of recurrence, and the cartilage used for reconstruction was found to be in the appropriate position allowing an adequate airway passage (Figure 3).

## 3. Discussion

The surgical options for thyroid cancer invading the larynx should be evaluated to decide the most appropriate choice for the patient. The other partial surgical techniques that can be applied to the presented patient include near total laryngectomy and tracheohyoidoepiglottopexy. The use of near total laryngectomy was not technically feasible because of the presence of a permanent tracheotomy, and the use of tracheohyoidoepiglottopexy was not technically feasible because the intact cricoid mucosa had been protected [6, 7].

Laryngotracheal invasion is a factor that worsens the prognosis in thyroid cancers. Although the surgical option is controversial, the technique that will be used is effective on quality of life. We think that surgical indication should not be established by the site of involvement in thyroid carcinomas invading the larynx, as in primary cancers of the larynx. While the site of the involvement is generally from the lumen to the cartilage in primary laryngeal cancer, it is from the cartilage into the lumen in thyroid cancer. This difference in the spread of tumors will lead to differences between the indications of surgical technique that will be established by the site of involvement. While cricoid and thyroid cartilage involvement in the presented case is an indication for total laryngectomy in primary laryngeal cancer, this allows partial laryngectomy in thyroid cancer invasion. We think that partial laryngectomy according to the involvement site and the appropriate reconstruction techniques should be used for thyroid cancer invading the larynx.
